# How to predict relapse in leukemia using time series data: A comparative in silico study

**DOI:** 10.1371/journal.pone.0256585

**Published:** 2021-11-15

**Authors:** Helene Hoffmann, Christoph Baldow, Thomas Zerjatke, Andrea Gottschalk, Sebastian Wagner, Elena Karg, Sebastian Niehaus, Ingo Roeder, Ingmar Glauche, Nico Scherf

**Affiliations:** 1 Institute for Medical Informatics and Biometry, Carl Gustav Carus Faculty of Medicine, School of Medicine, TU Dresden, Dresden, Germany; 2 AICURA Medical GmbH, Berlin, Germany; 3 National Center of Tumor Diseases (NCT), Partner Site Dresden, Dresden, Germany; 4 Max Planck Institute for Human Cognitive and Brain Sciences, Leipzig, Germany; Qatar University, QATAR

## Abstract

Risk stratification and treatment decisions for leukemia patients are regularly based on clinical markers determined at diagnosis, while measurements on system dynamics are often neglected. However, there is increasing evidence that linking quantitative time-course information to disease outcomes can improve the predictions for patient-specific treatment responses. We designed a synthetic experiment simulating response kinetics of 5,000 patients to compare different computational methods with respect to their ability to accurately predict relapse for chronic and acute myeloid leukemia treatment. Technically, we used clinical reference data to first fit a model and then generate de novo model simulations of individual patients’ time courses for which we can systematically tune data quality (i.e. measurement error) and quantity (i.e. number of measurements). Based hereon, we compared the prediction accuracy of three different computational methods, namely mechanistic models, generalized linear models, and deep neural networks that have been fitted to the reference data. Reaching prediction accuracies between 60 and close to 100%, our results indicate that data quality has a higher impact on prediction accuracy than the specific choice of the particular method. We further show that adapted treatment and measurement schemes can considerably improve the prediction accuracy by 10 to 20%. Our proof-of-principle study highlights how computational methods and optimized data acquisition strategies can improve risk assessment and treatment of leukemia patients.

## Introduction

Myeloid leukemias are characterized by aberrations affecting the proliferation and maturation of myeloid progenitor cells, leading to the progressive displacement of functional blood cells by immature and dysfunctional *leukemic* cells. Depending on the time scale of the displacement process, myeloid leukemias are further divided in chronic and acute leukemias.

Patients with chronic myeloid leukemia (CML) typically carry a disease-specific chromosomal translocation forming the *BCR-ABL1* fusion gene [1–4]. Tyrosine kinase inhibitors (TKI) have been established as a targeted therapy leading to molecular remission in most patients under continuous drug administration [5]. Molecular monitoring of disease-specific *BCR-ABL1* mRNA in peripheral blood is the established strategy to quantify the leukemic burden under ongoing therapy. Current therapeutic challenges include the cessation of TKI treatment, upon which about 50% of CML patients develop a molecular recurrence and do not maintain treatment-free remission [6–8].

Acute myeloid leukemia (AML) is a highly heterogeneous disease with a variety of mutational profiles involved [9]. Commonly, a cyclic induction therapy with cytotoxic drugs such as cytarabine and anthracyclines aims to achieve sustainable remission, while a subsequent consolidation therapy supports the maintenance of the remission status. Molecular detection of mutated oncogenes or their transcripts is increasingly used to monitor leukemic burden in treated AML patients and can help to prospectively identify patients at the onset of disease recurrence [10, 11].

Disease recurrence after treatment-induced remission is a significant risk for all leukemia patients. Although the reappearance of CML after TKI cessation can be targeted well by restarting the treatment, physical and psychological side effects of retreatment can be minimized if a prospective identification of ineligible patients can be achieved. AML relapse usually occurs after completion of intensive chemotherapy treatment [12] and is associated with a poor prognosis [13]. In those case, the ability to prospectively predict the risk and timing of relapse or molecular recurrence is of highest importance to optimize and adjust the individual treatment strategy.

Currently, treatment decisions are based on the recommended risk stratification schemes. Those risk assessments are commonly based on *static* measurements from single time points, often at diagnosis [14, 15]. In contrast, treatment response dynamics, such as the speed of initial remission, are only rarely evaluated for risk stratification [16]. However, it was shown that molecular disease dynamics indeed correlate with therapy response and future relapse occurrence [17–22]. We reason that the direct integration of molecular response dynamics in the form of time-series data, which are increasingly available from standard disease monitoring, is a crucial element to improve the patient-specific risk stratification.

Assessing this question from a technical point of view, there are several, conceptually different approaches to integrate time-series data from molecular disease monitoring into an improved risk assessment. It is so far not clear how well these approaches are suited for time course data of hematological malignancies, and what their particular strengths and weaknesses are in this context. In order to address this question, we study three methods representing typical examples of the methodological spectrum:

- *Mechanistic models* (MM) describe the molecular disease dynamics as a functional consequence resulting from the interaction between relevant system components (such as cell types, drugs, cytokines etc.). They are commonly implemented as systems of ordinary differential equations (ODE) or as stochastic models. While some model parameters might be directly measurable, other model-specific parameters are obtained by optimally fitting the simulated time course to the available patient data. Evolving the model further in time allows to simulate the expected future behavior. Although MMs require considerable expert knowledge about the underlying mechanisms, the results of these models are readily interpretable as the model parameters typically carry explicit biological meaning.
- On the other end of the spectrum, deep learning approaches [23–25] use generic *neural network models* (NN) to adapt them on a training data set for which time-series data and the corresponding future behavior is known. Roughly speaking, the NN implicitly identifies characteristic features within the time course data that correlate with future outcomes. Those methods require no *a priori* knowledge about the underlying mechanisms, but they are not suitable to directly interpret underlying biological mechanisms. Moreover, the training of NN requires a sufficient amount of annotated data.
- Classical statistical models like logistic regression classifiers can be used to correlate characteristic, predefined features of the time course data (such as speed of remission or remission level) with the known outcome. Such statistical models are summarized as *generalized linear models* (GLM) [26]. Herein, prior knowledge about general treatment dynamics is directly incorporated as an explicit feature of the GLM, while no understanding of the underlying biological mechanisms is required. Although GLMs are typically easier to interpret than neural networks (as the influence of parameters on the prediction can be assessed [27]) this probabilistic approach does not allow for explicit mechanistic interpretations as it is the case for MMs.

**Fig 1:**
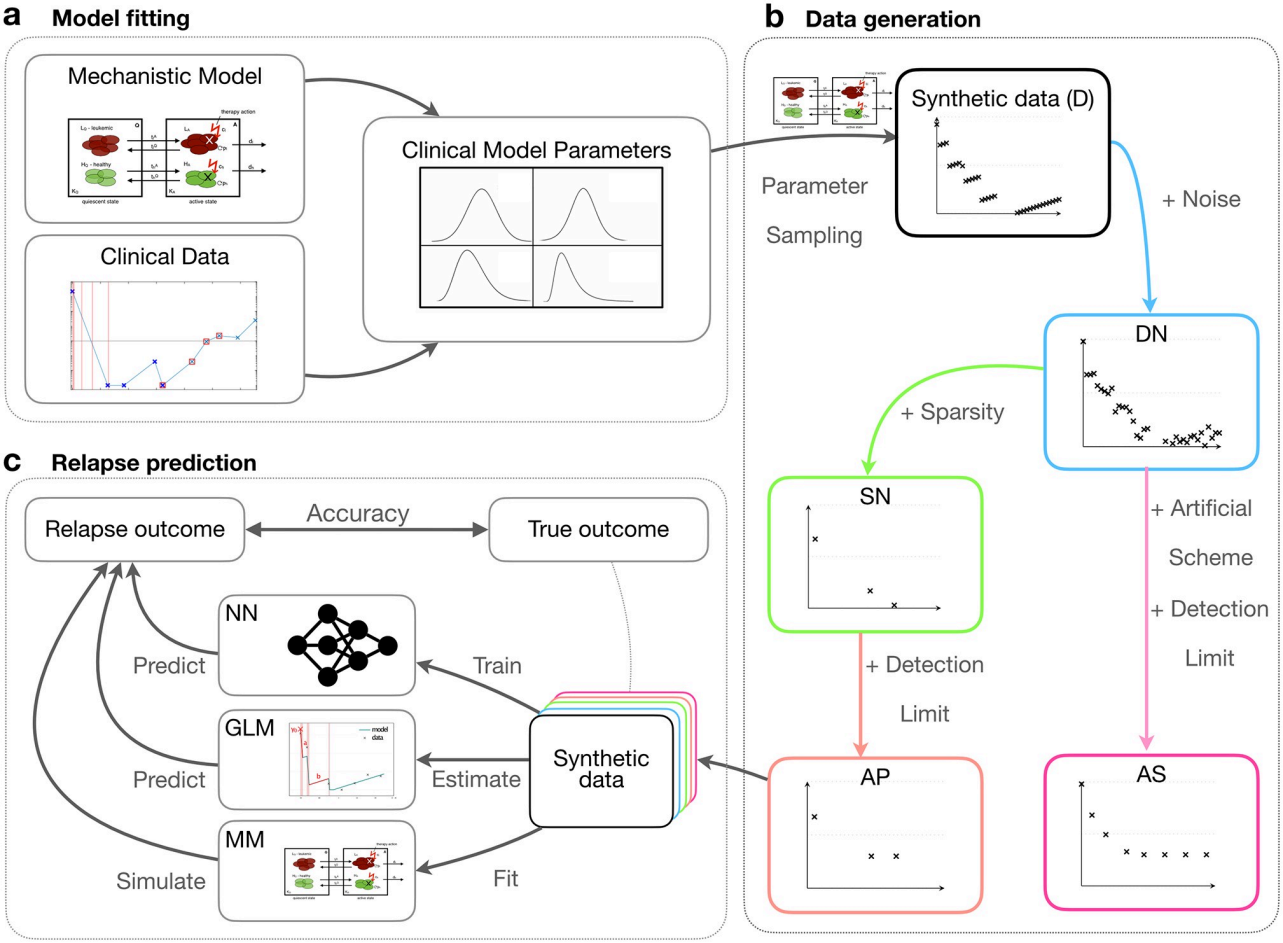
Conceptual overview of our methodological approach. (a) We developed mathematical models for both AML and CML from mechanistic and empirical knowledge [20, 28]. The models are first fitted to actual patient data to obtain realistic parameter distributions. (b) We sampled from these empirical parameter distributions to simulate dense, synthetic data (D). We gradually reduced the data quality to mimic actual clinical measurements by introducing noise (dense-noisy, DN), introduce sparsity (sparse-noisy, SN) and a minimum detection limit (artificial patient data, AP). Additionally, we introduced a more informative scheme (artificial scheme, AS), in which the temporal measurements are optimally spaced (AML) or a period of reduced treatment dose precedes therapy cessation (CML). (c) We systematically compared the performance of our mechanistic model (MM), a generalized linear model (GLM) and a neural network (NN) to predict the outcome (relapse/no relapse) of our virtual patient data with varying quality.

In this work, we systematically compare these three methods. In particular, we study the influence of data size, sampling density and measurement error on their prediction accuracy. As available data sets of relevant molecular time courses for AML and CML are currently limited, we first generate an artificial patient cohort (*synthetic data*) using different established mathematical models of those diseases [20, 28] (Fig 1). This artificial data set closely mimics the features of a smaller sample of real patient time courses, while the number of measurements and the particular noise level can be varied systematically and consistently. Based on this reference simulations, we are further able to suggest alternative disease surveillance schemes that may enhance the predictive power.

## Materials and methods

### Mechanistic models

To generate the synthetic data, we used two of our recently published mechanistic models for AML [20] and CML [28], both implemented as systems of ordinary differential equations (ODE). For the AML scenario, four ODEs are used to describe both leukemic and healthy stem cells. Two out of 11 model parameters are optimized to account for patient-specific differences in the disease characteristics, while the others were chosen to account for the general treatment dynamics. For the CML models, three ODEs represent active and inactive leukemic cells plus a population of interacting immune cells. In this case, we estimate 7 of 13 model parameters to optimally describe a patient’s response. Details of the model setup are provided in the S6 and S7 Figs.

### Patient data

For the generation of a set of realistic parameters, we fitted the respective mechanistic model to previously published time course data reflecting the patient’s leukemia remission during and after therapy. In particular, we used the time courses of 275 NPM1-mut AML patients, in which the level of NPM1-mut/ABL abundance is used as a measure of leukemia load (median follow-up time of 10 months, the median number of 5 measurements [20]). Furthermore, we integrated data sets from 21 CML patients reflecting both their BCR-ABL1/ABL1 remission levels under TKI therapy and after therapy cessation (median follow-up time of 84 months, the median number of 28 measurements [28]). Examples of model fits to patient data, and the mean absolute error for each fitted patient can be found in S1 Fig.

### Parameter fitting

Both, the AML and the CML model are initially fitted to the available patient data. Technically, we vary possible configurations of free parameters of the model such that the difference to the data is minimized (measured in terms of the sum of squares of the residuals on the logarithmic axis). While a simple optimization routine (*sequential quadratic programming*) is sufficient for the AML models, we apply a genetic algorithm combined with a gradient-based method for the CML scenarios which is better suited to avoid local minima. For further details we refer to the S1 File.

The same optimization routines are applied when the MMs are fitted to the artificial reference data for which we can tune data density and measurement noise (see below).

### Generation of artificial data

To generate artificial patient data, we take random samples from the sets of parameters that were initially derived from fitting the mechanistic models to the available patient data.

In the case of AML, it was sufficient to randomly sample new parameter combinations from the set of empirically observed parameters plus adding a small, normally distributed variation to prevent the generation of identical duplets (see S1 File). For the treatment regime (namely the number and timing of induction cycles) we sampled one particular clinical chemotherapy schedule which we observed in the given patient data. Only artificial patients that reached remission (i.e. leukemic burden fell below the threshold of 1%) were included in the data sets. Using this parameterization and the corresponding schedules, we simulated artificial time courses of 24 months length. In analogy to the clinical situation, AML relapse is assigned if the fraction of leukemic increases above the threshold of 1% within 2 years after treatment start.

For the corresponding artificial CML time-courses, we sampled the seven model parameters from the distribution of empirical estimates in the available data basis under the condition that their mutual correlations are maintained (for details see S1 File). The time of therapy cessation was sampled based on kernel density estimates from the cessation time of the given patients (avg of 92 months with a standard deviation of 28.2 months). This information was then used in de novo forward simulations to generate artificial time-courses of varying duration until treatment stop plus 10 years thereafter. CML recurrence was defined as leukemia abundance > 0.1% (corresponding to BCR-ABL1/ABL1 = 0.1%, MR3).

In order to study how the data quality influences the prediction quality, we generated the following five reference data sets for both disease scenarios (examples in S2 and S3 Figs):

- Dense data (D): with weekly (AML) or monthly (CML) exact measurements, respectively.
- Dense-noisy data (DN): where white noise was added to each measurement, according to the noise level found in the given clinical patient data.
- Sparse-noisy data (SN): generated from the DN data set by reducing the number of data points to reflect the measurement frequency in clinical patient data.
- Artificial-Patient data (AP): by adding a detection limit to the SN data as found in the clinical patient data.
- Artificial scheme data (AS): Similar to AP data but using an improved sampling scheme compared to the clinical patient data. For AML measurements are made at the end of each chemotherapy cycle and every six weeks afterwards. For CML, the treatment dose is reduced to half of the usual dose 12 months before therapy cessation with frequent measurements during this period.

Using this synthetic reference data, we use the following setup to evaluate the correctness of predictions. For AML, all measurements from the initial treatment phase to 9 months after diagnosis are provided to the three methods and a corresponding relapse prediction within the subsequent 15 months is derived. For CML, we use all measurements up to the treatment stop to predict whether a patient will present with disease recurrence within ten years thereafter. The long timespan has been chosen to reflect the slow evolution of CML. To obtain the corresponding model predictions from the MM, we fitted the model parameters to the initial time course data (see above) and then simulated the future behavior using the fitted model parameters for each dataset individually. In contrast, both GLM and NN are optimized using a 10-fold cross validation on labelled data sets for which the respective outcome of relapse occurrence is provided.

### Explicit features of time series for GLM analysis

As the Generalized Linear Model, we use a logistic regression classifier. The model uses explicit features that describe characteristics of the time-course data. We took the two characteristics of AML time-courses defined in our previous work [19]: the elimination slope *α*, describing the speed of decrease of leukemic burden over the time of treatment and the lowest measured leukemic burden after treatment *n*. In this work, we further added three additional features obtained from a segmented regression approach: the leukemic burden at diagnosis (*y*_*0*_, the following decreasing slope during the times of treatment (*a*) and the increasing slope of the leukemic burden in between treatment cycles (*b*) (S4A Fig).

For CML, we defined seven features from fits of a bi-exponential function that described the decrease of the leukemic burden after treatment start. These features include the bi-exponential parameters (*A*, *α*, *B*, *β*), the corresponding deviation of the fit and the data (σ), the cessation time and the BCR-ABL1 value before cessation or half dose. For the AS data, we expand these features with the behavior of the leukemic burden during the time of dose reduction including linear function parameter (*γ*), the deviation during half dose (*C*) and the last measured value before cessation (S4B Fig).

### Neural network

NN were only trained on the raw time course data with no explicit features provided. To predict the occurrence of relapse, we used a bidirectional Long-short-term-memory (LSTM) network as a default architecture to handle sequence data with varying length. The model consists of a bidirectional LSTM layer followed by a fully connected feature extractor and a binary classification output. We use the respective cross-entropy loss to train the network. We implemented the network in Python using the Keras library [29]. To get a robust estimate of the model performance, we conducted 10 training runs on the same dataset and chose the network with the highest validation accuracy. We then did 10-fold cross-validation for the entire experiment to assess the average and the variability of the results. Further details about the network architecture and training can be found in the S1 File.

### Accuracy

We use the traditional definition of accuracy as the ratio of the number of correct predictions over the total number of predictions: $acc=\frac{\#correct}{\#total}=\frac{TP+TN}{TP+FP+TN+FN}$ where TP, TN, FP, and FN are true positives, true negatives, false positives and false negatives respectively.

## Results and discussion

### Artificial patient data provide a suitable basis to systematically analyze the performance of predictive, computational models

We apply two mechanistic, mathematical models to simulate the dynamics of AML and CML [20, 28] thereby creating sets of artificial response data. To make sure that the artificial data resemble real patient time-courses as closely as possible, we fitted the models to respective data sets obtained from 275 AML patients carrying a traceable NPM1-mutation (consisting of a total of 1567 measurements quantifying the relative amount of NMP1-mut transcript [20] over time on a log10-scale) and 21 CML patients (with in total 478 measurements [28] quantifying the relative amount of BCR-ABL transcripts over time on a log10-scale). We report on the overall fitting quality in S1 Fig The fitted model parameters are used to simulate synthetic time courses (Fig 1a and 1b). To assess the influence of data quality, we gradually degraded the fully sampled, noise-free time series. We used estimates of the measurement frequencies and measurement errors obtained from the patient data to adjust the corresponding sampling density and noise level for the synthetic data (see S1 File). In total, we created four different datasets with 5000 time-courses from each model to systematically study the influence of data quantity and quality: (i) a dense (D) data set consisting of weekly (AML) or monthly (CML) measurements of the leukemic burden free of any measurement error. (ii) For the dense-noisy (DN) data we added a normally distributed “technical” noise (see S1 File) to all data points of D to match the measuring error (AML) or the residuals observed between real data and their corresponding model fits (CML). (iii) In a third step, we reduced the total number of measurements per patient, creating a sparse-noisy (SN) data set that matches the measurement frequency in the real data. (iv) Finally, to make the data as realistic as possible, we also added a detection limit for very low measurements, called artificial patient (AP) data. Example time courses for all data sets can be found in S2 and S3 Figs.

To verify that the created artificial patient data (AP) sets are indeed similar to the real patient data, we derived characteristic features to quantitatively compare them. Those characteristic features refer to typical time scales and remission levels of the patient’s response (see S4 Fig, Materials and methods). The features are computed separately for the AP data and the given patient data. The visual comparison in S5 Fig indicates that the median values of the characteristic features are very similar between AP and real data. It appears, that especially for the case of CML, the synthetic data sets yield a larger variance compared to the real data. A closer look at the data reveals that this is effect, at least partially, results from a sampling effect, as the variance measurement is only based on a small data set (n = 21) of real patients.

### Data quality has a strong influence on prediction accuracies, but the drop in performance considerably differs between models and use-cases

Similar to the clinical presentation, we classified the synthetic time-courses as whether they show a relapse or not. For both CML and AML, we define disease recurrence by an increase of the leukemic burden (measured in terms of relative transcript abundance) within a predefined period above a given threshold (AML: leukemic burden increasing > 1% after treatment termination; CML: leukemic burden > 0.1% for at least one month).

We then systematically compared the accuracy of relapse predictions between the three general methods (namely MM, GLM, NN). To do so we provide each method with data from the initial treatment phase and compare the resulting predictions with the ground truth from the artificial data sets. For AML, we provide all measurements from the initial treatment phase until 9 months after diagnosis and derive a corresponding prediction on whether a relapse is expected within the subsequent 15 months. For CML, we use all measurements up to treatment stop to predict whether a patient will present with disease recurrence within ten years thereafter. We use the following strategy to derive predictions for the three methods: MM: fitting the mechanistic model to the initial treatment data only and further simulating the future time course, GLM: feeding the explicit features of the initial time-course (see Materials and methods) into a GLM classifier and NN: using an end-to-end learning approach with a neural network model applied to the initial time-course, which has been trained previously on an annotated reference data set (Fig 1c).

**Fig 2:**
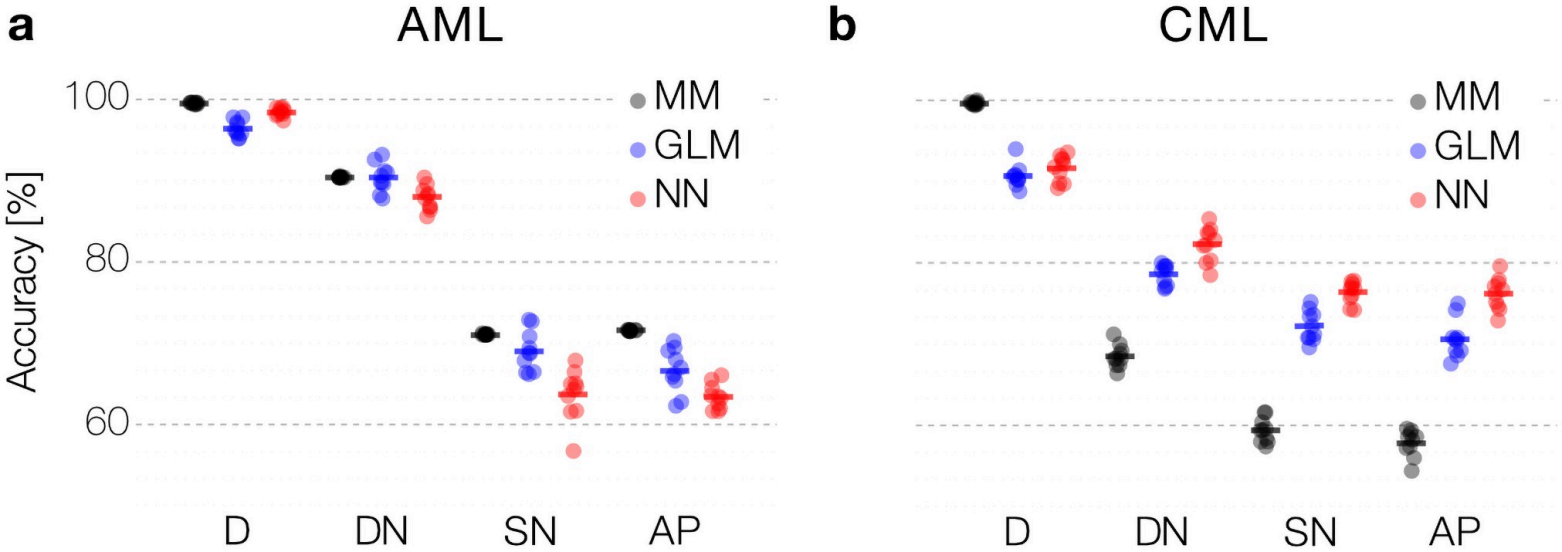
Prediction accuracy across data quality and computational models. (a, b) Comparison of performance between mechanistic model (MM), generalized linear model (GLM) and neural network (NN) to predict relapse in synthetic data for AML (a) and CML (b) using 10-fold cross-validation. Data quality gradually decreases from fully sampled, noise-free data (D), to noisy (DN), sparse and noisy (SN), and artificial patient data (AP) (see main text for details).

Next, we analyzed how well the different approaches (MM, GLM, NN) can predict the outcome for the artificial patient data and how model performance changes with varying data quality (Fig 1b and 1c, and Experimental Procedures). The results of the 10-fold cross-validation of the model performance are depicted in Fig 2. As expected, the prediction accuracy (see Materials and methods) declines for all approaches when the data quality decreases. We point out that the decrease in data quality differs between use-cases and models. In the case of AML, the introduction of sparsity leads to a relatively sharp drop in model performance. This drop illustrates the strong dependency on the number of measurements per time series: as in the given patient we only have a median of 4 measurements in the SN and AP data, compared to 39 weekly measurements in the dense data set (D) set. In line with this argument, we observe a more gradual decline in performance when comparing the effect of introducing noise and sparsity in the CML case. Here, we face a median of 25 measurements in the SN and AP data, compared to 93 monthly measurements in the dense data (D).

Interestingly, the difference in model performance is not consistent across the two use-cases. For the sparser AML data, all models perform similarly on the dense (D) and noisy data (DN). However, when introducing more sparsity into the data, a mechanistic model performs more robustly than the generic NN model (a difference in the accuracy of 6.3 and 7.4 percentage points for the SN and AP) and the GLM model performance is in between MM and NN. This result reflects the importance of introducing prior knowledge (or inductive bias) when dealing with very few data (Fig 2a).

We observe a different situation in the CML case. Here, the prediction accuracy for the mechanistic model drops down substantially more compared to the statistical GLM model and the generic NN when data quality decreases (a difference in accuracy between MM and NN of 19.7% for SN and 19.8% for AP, respectively). We recall that the noise-free data (D) was generated by the very same mechanistic model (compare Fig 2b). The high prediction accuracy for this data indicates that the correct (generative) MM can truly be identified. However, given the higher number of free parameters (n = 7) in the CML case, a reduction of data quality (either resulting from noisy or sparse measurements) more strongly affects the identifiability of the correct MM, while the GLM and the NN appear more robust.

Focusing on the artificial patient samples (AP), which best mimic the available patient data sets, the suggested models reach an accuracy of up to 70% (compare Fig 2a and 2b). These findings shows that predictive computational methods can indeed support risk assessment in myeloid leukemias based on nontrivial patterns in time series data obtained during treatment. However, the resulting prediction accuracy might not adhere to the expected standards for clinical decision support. Our systematic analysis shows how data characteristics, in particular the measurement schedule, effects the performance. Data scarcity and limited accuracy of available measurements per patient appears as a limiting factor for the overall prediction accuracy for relapse occurrence. Given those constrains on the data side, we are skeptical that structural changes to the computational methods (e.g. by refining the neural network architecture) can substantially improve the overall performance. However, below we outline the potential in optimizing the measurement process to yield more informative sampling schemes.

### Refined measurement and treatment schemes lead to improved prediction accuracies

**Fig 3:**
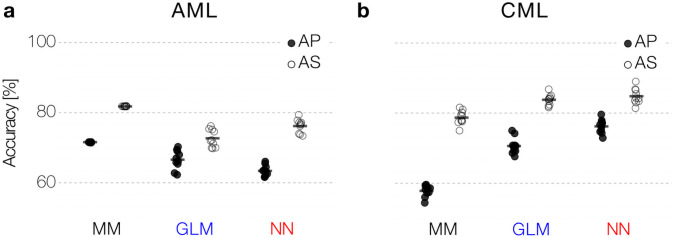
Dedicated measurement schemes. (a, b) A dedicated measurement scheme (AS) improves prediction performance with the same number of data points for all models compared to the AP data both for AML (a) and CML (b) data.

We demonstrated that a significant limitation for the prediction accuracy results from the sparsity of the available data, in particular for the case of AML. Here, molecular diagnostics and especially bone marrow aspirates are limited resources in the clinical setting. As only increasing the sampling frequency is not an option in many cases, we wondered whether an optimized timing of the measurements could lead to better predictions while the overall number of measurements remains the same. To investigate this question, we created an additional set of artificial patients (AS) with consistent measurement intervals during the nine-month treatment period (i.e. the first day of each therapy cycle and every six weeks during the treatment-free phase). This typically results in 4 to 8 (median = 7) measurements per patient, which is only a moderate increase to the reported median of 5 measurements in the clinical sample. Fig 3a indicates that for this amended sampling regimen, we can already increase the accuracy of all prediction approaches (MM and NN by up to 12%, less pronounced for GLM). This finding strongly suggests that an adapted sampling scheme can considerably contribute to better relapse predictions, e.g. using methods from an optimal experimental design [30–33].

Owing to the establishment of regular BCR-ABL measurements in TKI-treated CML patients, available time courses are usually sufficient to monitor treatment response and remission status. It is still controversial, to which extend treatment free remission correlates with the observed time course of initial response [22, 28]. However, results from the DESTINY trial [34] suggest that dynamics of BCR-ABL increase during TKI dose reduction correlates with the remission status after treatment cessation [18]. The DESTINY trial differs from other TKI stop trials as patients in molecular remission reduced their TKI dose to 50% of the original dose for 12 months before TKI was finally stopped [34]. Motivated by this study, we simulated a corresponding data set in which a 12-month dose reduction is explicitly added to the model simulation (AS dataset). Training the prediction approaches to explicitly integrate this additional 12 month perturbation period, we found a substantial increase in the prediction accuracy of up to 19.1% (Fig 3b). We argue that probing the system’s response to perturbation (such as dose reduction) provides additional information about control mechanisms that cannot be obtained from ongoing monotherapy [16, 18, 28].

Our analysis demonstrates that optimized measurement schedules or systematic treatment alterations can substantially improve the accuracy of relapse predictions.

## Conclusions

We showed that qualitatively different computational approaches, ranging from machine learning approaches to mechanistic models, are in principle suited to support relapse prediction based on time-series data of leukemia remission levels. To this end, we employed simulated time course data generated by mechanistic mathematical models, which we previously developed to describe disease and treatment dynamics in CML and AML. It is the advantage of this approach that we obtain highly controlled, although idealized, remission curves as a reference set from which we can abstract different levels of sampling density and measurement error. The simulated data allows us to refer to the *ground truth* of the underlying generative model. Using this artificial reference data, we could demonstrate that data quality in terms of measurement frequency and measurement error has a more substantial influence on the accuracy of the prediction than the employed prediction method, which is particularly evident in the AML data. Our results for the CML case indicate that fitting a more complex mechanistic model (in terms of the number of model parameters) to noisy data yields a greater uncertainty compared to a statistical predictor like a GLM or a NN.

Our analysis illustrates that generic methods, such as NN work well for the prediction of disease recurrence if frequent measurements are available (as in the CML data). For diseases with sparse measurements and limited data on the other hand (exemplified in the AML data), neural networks (and representation learning in general) is less suited for identifying the critical factors underlying the disease dynamics. In such cases, it is beneficial to incorporate prior knowledge to yield better predictions using either mechanistic models of the disease, if available, or statistical approaches based on explicit (phenomenological) features. In our current study, we used a long-short-term-memory (LSTM) NN as the standard approach for analyzing sequential data. An interesting next step is to assess if more complex neural network models [35, 36] can even improve the LSTM results, although we suspect that data quality is the major limiting factor.

Overfitting is a known problem of all machine learning approaches and applies to both the GLM and the NN method we presented. In order to minimize this risk, we applied a 10-fold cross validation which was also used to estimate the variation of the estimated accuracies. Our general approach is limited by the generation of time course data from generative models which intrinsically do not reflect “unexpected” behaviors. As long as the true data basis of clinical time courses is limited, only the additional consideration of alternative generative models could help to address this issue.

Regardless of the exact choice for a predictive computational method, our study indicates that the optimization of measurement schemes and clinical protocols is a promising strategy to improve the overall prediction accuracy without necessarily requiring more measurements per patients. In our predictions for AML recurrence, we could reach a level of accuracy of about 80% for the prognosis of relapse occurrence within two years after diagnosis. This result would already exceed the prediction accuracy for relapse-free survival after 12 months in the study by [15]. As our results are based on synthetic data which most likely does not reflect the full heterogeneity that could be seen in larger patient data sets, our comparison should be treated with caution and needs to be validated using independent clinical data obtained in a comparable context. Still, our findings indicate that standardized measurement schedules add critical leverage to improve the ability for predicting relapse no matter what computational methods are used. Our artificial measurement schemes indicated a clear improvement, while we did not even apply formal optimization criteria to obtain most suitable regimes that maximizes accuracy while minimizing the number of measurements. This finding opens a clear perspective for future research on optimized measurement strategies that balance a maximized gain of information from clinical data with an economical use of resources. We argue that such refined schedules can contribute to reaching a level of prediction accuracy, which indeed supports clinical decision making.

In this work, we focused on the accuracy of relapse prediction employing three different, prototypic computational approaches working on time-series data. However, their implementation in a decision-making context also requires an intuitive understanding of how the method works. Although NN do not require any prior knowledge and can achieve excellent prediction accuracies, it is not trivial to identify which aspects of the data are causative for a particular prediction [37, 38]. In other words, the "black box" nature of NN does intrinsically not reveal the key features of the data on which a decision is based. There is a general, ongoing scientific discussion whether this intrinsic limitation of NN should prevent its application for particular questions, especially in health care [39, 40]. Currently, decision-makers and regulatory authorities hardly consider such methods for integration into clinical routines, although this might change in the future. Orthogonal developments in the field of “explainable AI” are currently pushing towards interpretability and the identification of causal relations between different system components [41–43]. As for now, MM represent the other side of the "interpretability spectrum" as they superimpose a principal understanding of the causal interactions onto the final observations. It appears tempting to favor this type of approach. However, it comes with other limitations: such models are highly specific and not easily transferable to other disease entities, and it cannot be guaranteed that all essential interactions are indeed mapped (compare [20]). The extent to which the non-representation of potential interactions effects the model predictions is hard to quantify and most likely highly disease specific. GLMs represent a middle ground and balance several aspects of NN and MM approaches. They can be helpful if detailed mechanistic knowledge is missing while important features of the response characteristics can readily be named, estimated and also interpreted. However, their overall performance depends strongly on the choice of those hand-crafted features and is also vulnerable to missing critical aspects.

The increasing availability of diagnostic methods to track molecular remission in different cancer types over extended time periods will establish a rich data source to explore further how this dynamic information can be correlated with the future course of treatment and disease [16]. Obtaining a systematic understanding of how different computational methods can be used to exploit this data is of crucial importance to provide usable predictions. Sufficient model validation within the particular domain is the prerequisite to integrate such computational models into decision making in a clinical context.

## Supporting information

S1 Fig Mechanistic model fit to patient data. (a) Example time-course of an AML patient (measured in terms of NPM1-mut abundance relative to reference gene ABL; blue dots) from start of chemotherapy at time point 0 until molecular relapse and the respective model fit (solid line; leukemic burden, rescaled by a factor 100 to match the clinical NPM1-mut/ABL ratios [20]). Red lines indicate time of chemotherapy administration. (b) Mean absolute error (MAE) for the fit of the mechanistic model to all 275 AML patients time-courses. (c) Example time-course of a CML patient (measured in terms of BCR-ABL/ABL abundance; black dots; triangles indicate undetectable BCR-ABL levels with the corresponding detection threshold) from start of TKI treatment at time point 0 until disease recurrence after treatment stop (grey region) and respective model fit (solid line). (d) MAE of all 21 fitted CML patients.
(TIFF)

S2 Fig Generation of the artificial AML data sets. We use a sample patient for which we obtain weekly and precise measurements, referred to as *dense* data (D). Adding a technical, normally distributed noise to each measurement on the log-scale, we obtain *dense-noisy* data (DN). *Sparse-noisy* data (SN) was generated from the DN data set, by reducing the number of data points to meet the measurement frequency in real patients. *Artificial patient* data (AP) is the data set most similar to the real patient data, which differs from the SN data set only by the inclusion of a detection limit (dashed red line), as it is found in the real data. *Artificial scheme* data (AS) is a data set, close to real data, with a measurement scheme, where measurements are made at the end of each chemotherapy cycle and every 6 weeks afterwards.
(TIFF)

S3 Fig Overview of artificial CML data sets. *Dense* data (D) was simulated with monthly exact measurements. *Dense-noisy* data (DN) was obtained by adding normally distributed noise to each measurement. *Sparse-noisy* data (SN) was generated from the DN data set, by reducing the number of data points to meet the measurement frequency in real patients. *Artificial-Patient* data (AP) is the data set most similar to the real patient data, which differs from the SN data set only by the inclusion of a detection limit, as it is found in the real data. *Artificial scheme* data (AS) is a data set, close to real data, with an additional 12-month period of half-dose TKI treatment (shown in grey).
(TIFF)

S4 Fig Derived features of the time-courses. (a) Features describing AML time courses: *y*_*0*_ the leukemic burden at diagnosis, *a* the decreasing slope during treatment cycles, *b* the increasing slope in treatment free intervals (where *y*_*0*_, *a* and *b* are obtained from a segmented regression approach), *α* the overall decreasing slope during treatment (shown as dashed line, separately fitted to the measurements) and *n* the minimal leukemic burden after treatment. (b) Features describing CML time courses: *A*, *B* and *C* being the intercepts of the straight lines fitted to the first and the second part of the bi-exponential approximation and to the increase of the leukemic burden during half-dose periods, respectively. *α*, *β* and *γ* are the respective slopes.
(TIFF)

S5 Fig Similarity of artificial patients and real patients. A distribution comparison of statistical parameters: Comparison of distribution of parameters describing the course characteristics between artificial patient data (AP, blue) and real data (RD, black). (a) Parameters characterizing AML response: a—decreasing slope during chemotherapy cycle, *b—*increasing slope during treatment-free periods, *y*_*0*_—initial burden on log scale, *α*—elimination slope, *n*—minimal measured leukemia burden after primary treatment. (b) Parameters characterizing CML response: the intercepts *A* and *B* (on a log scale) as well as the slope parameters *α* (on log scale) and *β*.
(TIFF)

S6 Fig Schematic overview of the mathematical model for AML. Both, leukemic *L* and healthy *H* stem cells can reversibly change between two states (according to the rates *t)*: the quiescent state Q with carrying capacity *K*_*Q*_ and the active state A with carrying capacity *K*_*A*_. Cells in A undergo proliferation with rate *p*, differentiation with rate *d* and are subject to chemotherapy with kill rate *c*.
(TIFF)

S7 Fig Schematic overview of the mathematical model for CML. Leukemic stem cells (LSC) can reversibly change between two states X and Y (according to the rates *p*_*XY*_ and *p*_*YX*_, respectively): X defines the quiescent, non-replicating cells, Y defines the active, proliferating cells. LSC in Y proliferate according to a logistic growth model with maximal proliferation rate *p*_*Y*_ and carrying capacity *K*_*Y*_. The TKI-effect is described by a constant rate *e*_*TKI*_ affecting the leukemic cells in Y. Immune cells in Z are activated by cells in Y (immune recruitment), following an immune window approach (see Supporting information). At the same time the immune cells kill proportional target cell in Y. Immune cells in Z are generated with rate *r*_*z*_ and decay with rate *a*.
(TIFF)

S1 File Supporting information. (PDF)
