# Supplementary figures and images for: How to predict relapse in leukemia using time series data: A comparative in silico study
Source: PLoS One. 2021 Nov 15;16(11):e0256585. doi: 10.1371/journal.pone.0256585 (PMC8592437; doi:10.1371/journal.pone.0256585)

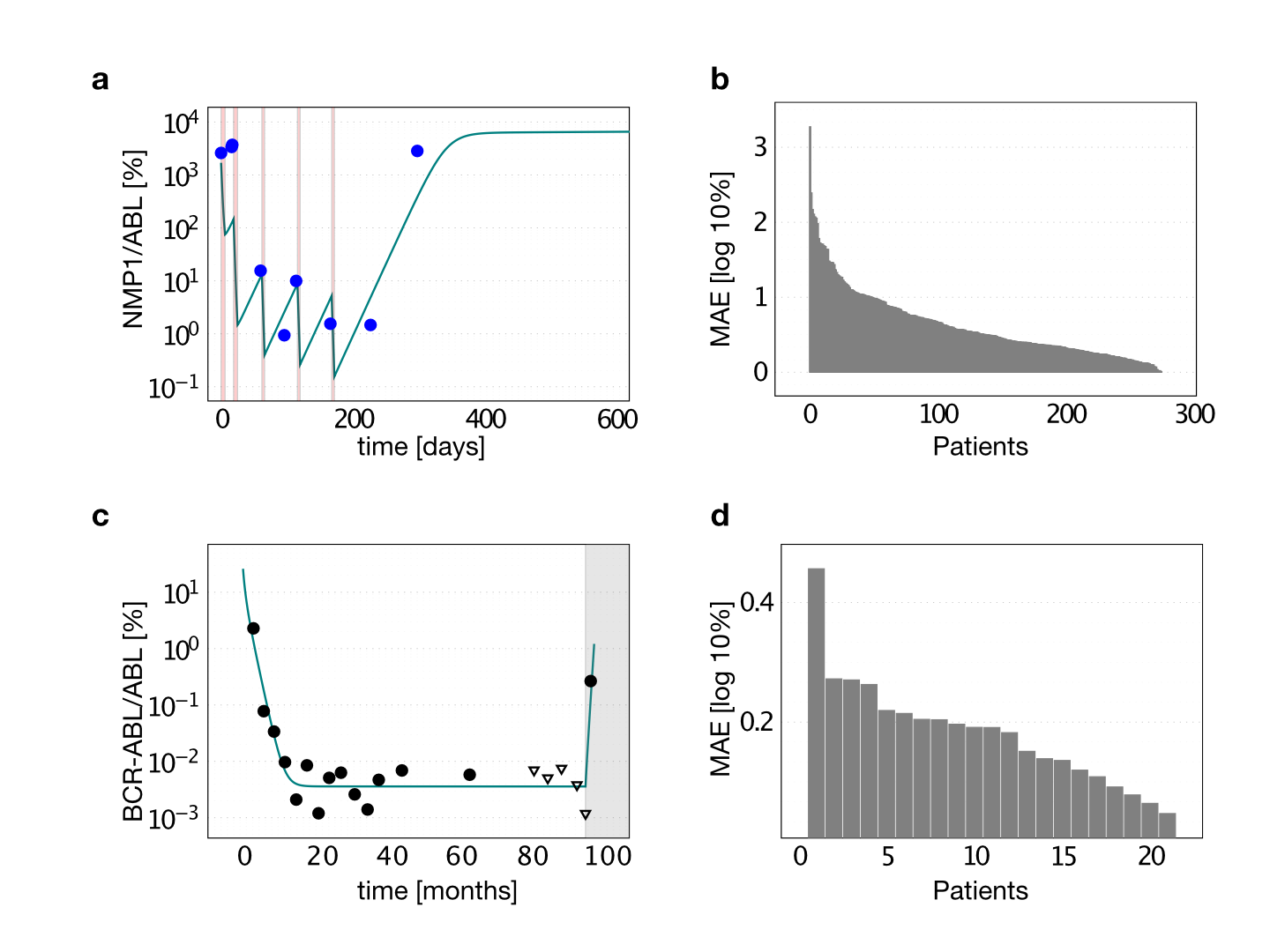

Supplement: S1 Fig — (a) Example time-course of an AML patient (measured in terms of NPM1-mut abundance relative to reference gene ABL; blue dots) from start of chemotherapy at time point 0 until molecular relapse and the respective model fit (solid line; leukemic burden, rescaled by a factor 100 to match the clinical NPM1-mut/ABL ratios [20]). Red lines indicate time of chemotherapy administration. (b) Mean absolute error (MAE) for the fit of the mechanistic model to all 275 AML patients time-courses. (c) Example time-course of a CML patient (measured in terms of BCR-ABL/ABL abundance; black dots; triangles indicate undetectable BCR-ABL levels with the corresponding detection threshold) from start of TKI treatment at time point 0 until disease recurrence after treatment stop (grey region) and respective model fit (solid line). (d) MAE of all 21 fitted CML patients. (TIFF) [file pone.0256585.s001.tiff]

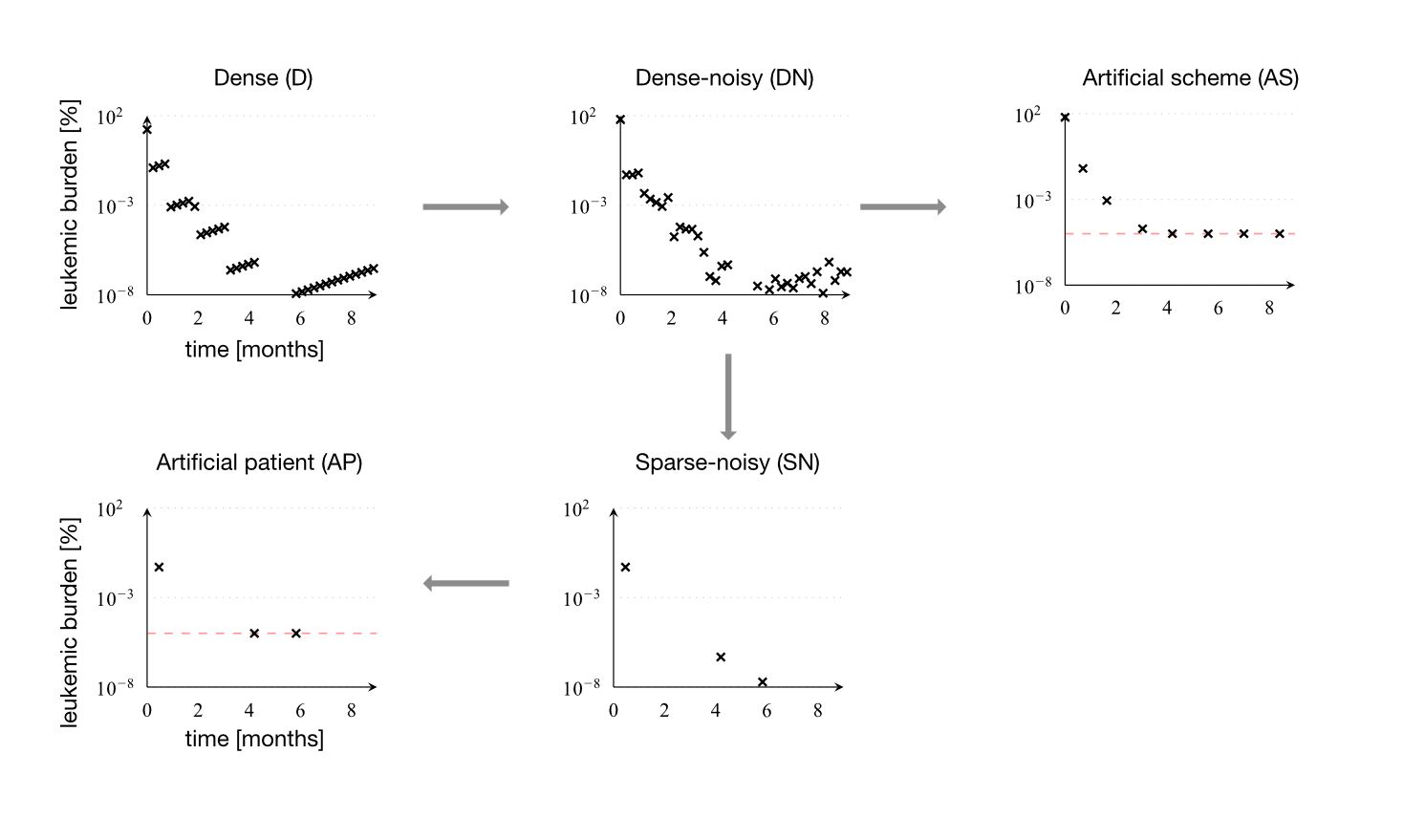

Supplement: S2 Fig — We use a sample patient for which we obtain weekly and precise measurements, referred to as dense data (D). Adding a technical, normally distributed noise to each measurement on the log-scale, we obtain dense-noisy data (DN). Sparse-noisy data (SN) was generated from the DN data set, by reducing the number of data points to meet the measurement frequency in real patients. Artificial patient data (AP) is the data set most similar to the real patient data, which differs from the SN data set only by the inclusion of a detection limit (dashed red line), as it is found in the real data. Artificial scheme data (AS) is a data set, close to real data, with a measurement scheme, where measurements are made at the end of each chemotherapy cycle and every 6 weeks afterwards. (TIFF) [file pone.0256585.s002.tiff]

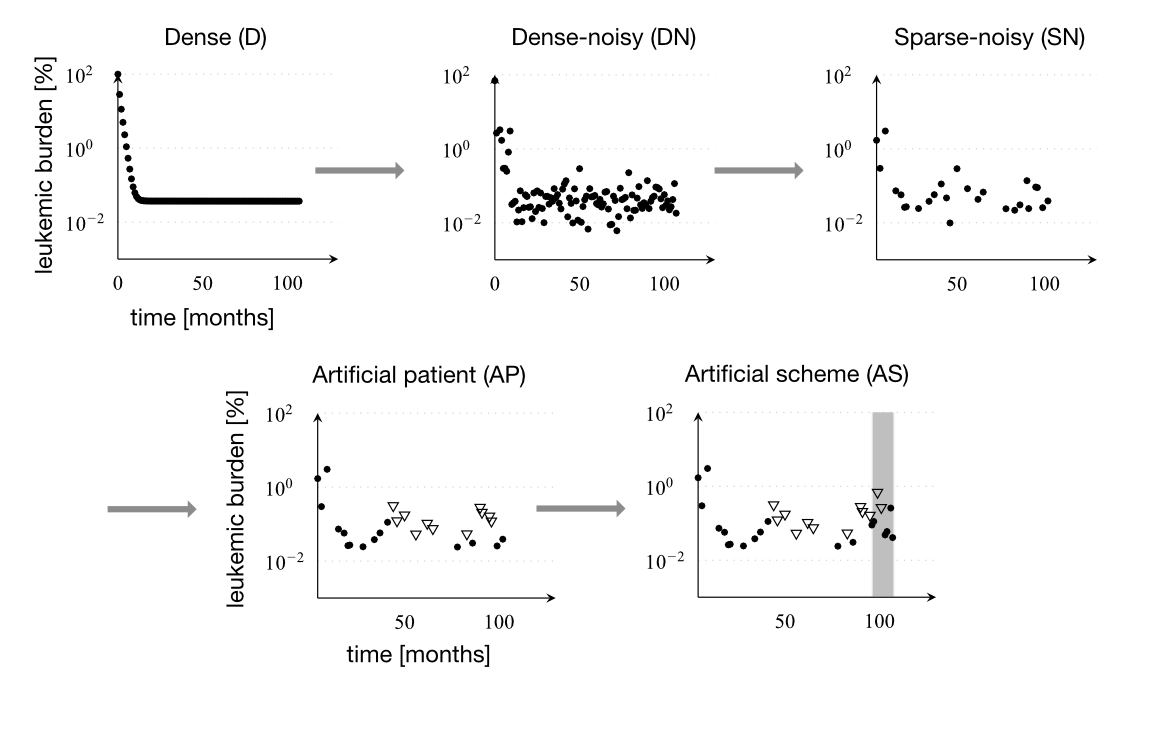

Supplement: S3 Fig — Dense data (D) was simulated with monthly exact measurements. Dense-noisy data (DN) was obtained by adding normally distributed noise to each measurement. Sparse-noisy data (SN) was generated from the DN data set, by reducing the number of data points to meet the measurement frequency in real patients. Artificial-Patient data (AP) is the data set most similar to the real patient data, which differs from the SN data set only by the inclusion of a detection limit, as it is found in the real data. Artificial scheme data (AS) is a data set, close to real data, with an additional 12-month period of half-dose TKI treatment (shown in grey). (TIFF) [file pone.0256585.s003.tiff]

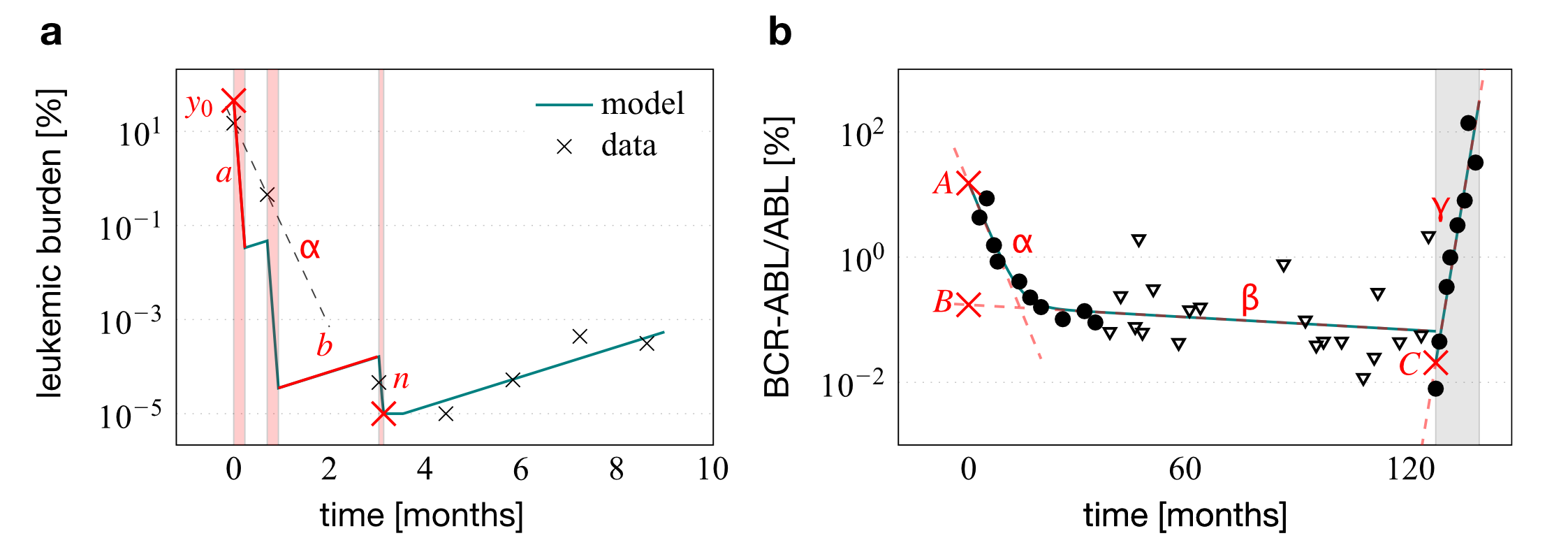

Supplement: S4 Fig — (a) Features describing AML time courses: y0 the leukemic burden at diagnosis, a the decreasing slope during treatment cycles, b the increasing slope in treatment free intervals (where y0, a and b are obtained from a segmented regression approach), α the overall decreasing slope during treatment (shown as dashed line, separately fitted to the measurements) and n the minimal leukemic burden after treatment. (b) Features describing CML time courses: A, B and C being the intercepts of the straight lines fitted to the first and the second part of the bi-exponential approximation and to the increase of the leukemic burden during half-dose periods, respectively. α, β and γ are the respective slopes. (TIFF) [file pone.0256585.s004.tiff]

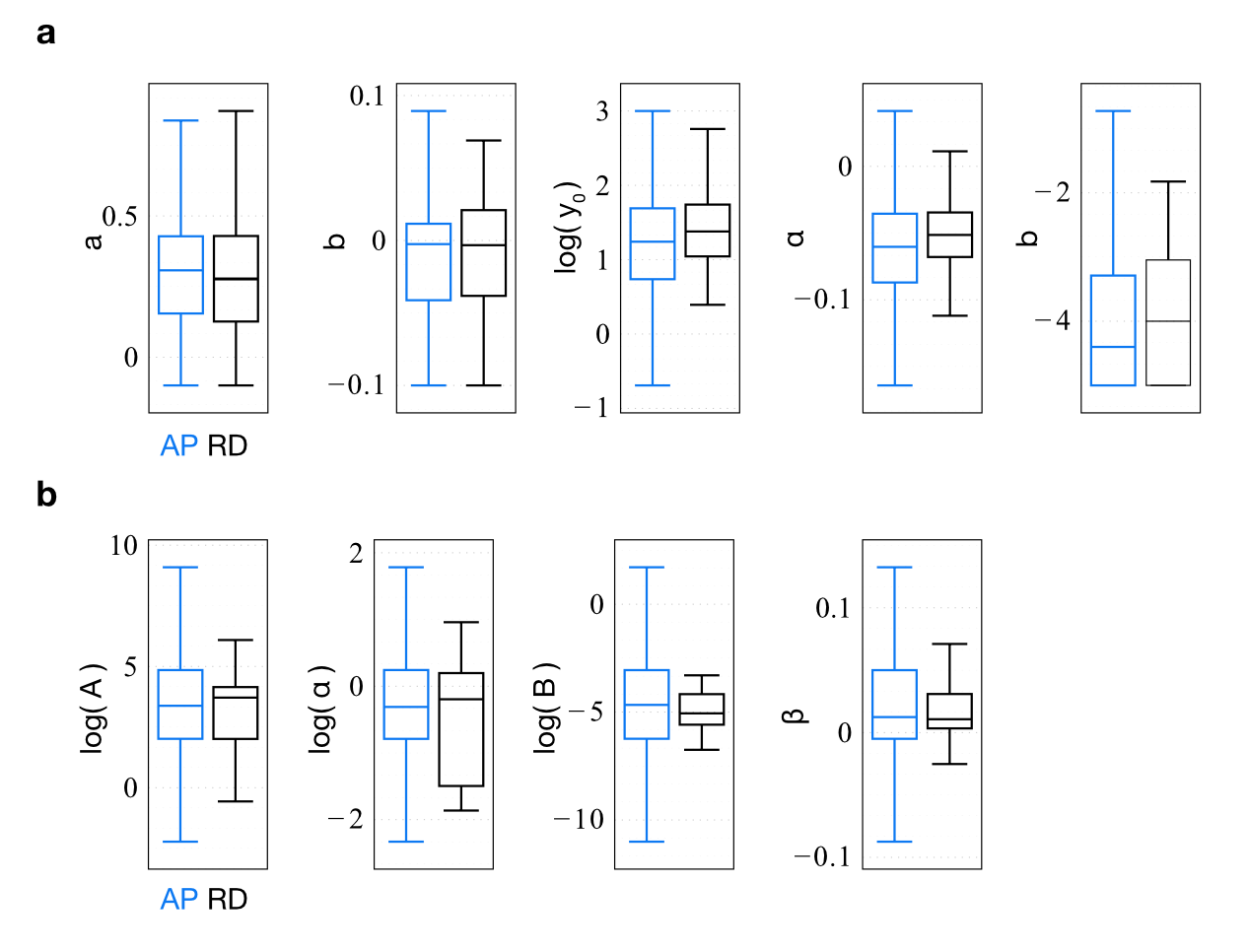

Supplement: S5 Fig — A distribution comparison of statistical parameters: Comparison of distribution of parameters describing the course characteristics between artificial patient data (AP, blue) and real data (RD, black). (a) Parameters characterizing AML response: a—decreasing slope during chemotherapy cycle, b—increasing slope during treatment-free periods, y0—initial burden on log scale, α—elimination slope, n—minimal measured leukemia burden after primary treatment. (b) Parameters characterizing CML response: the intercepts A and B (on a log scale) as well as the slope parameters α (on log scale) and β. (TIFF) [file pone.0256585.s005.tiff]

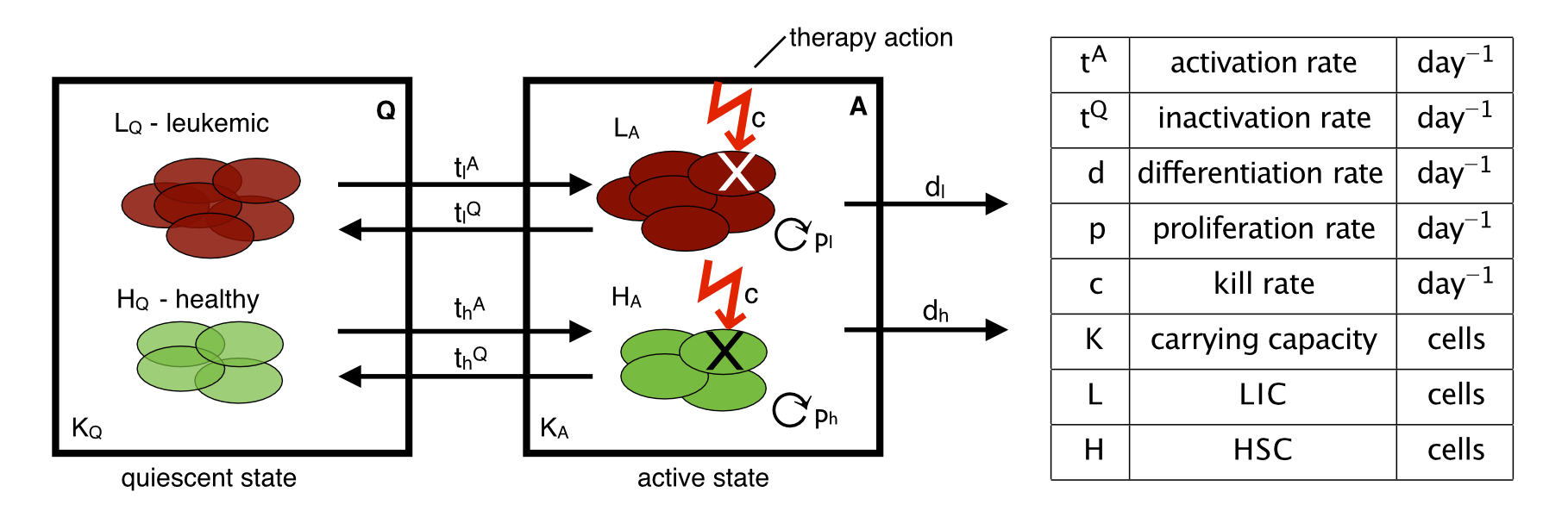

Supplement: S6 Fig — Both, leukemic L and healthy H stem cells can reversibly change between two states (according to the rates t): the quiescent state Q with carrying capacity KQ and the active state A with carrying capacity KA. Cells in A undergo proliferation with rate p, differentiation with rate d and are subject to chemotherapy with kill rate c. (TIFF) [file pone.0256585.s006.tiff]

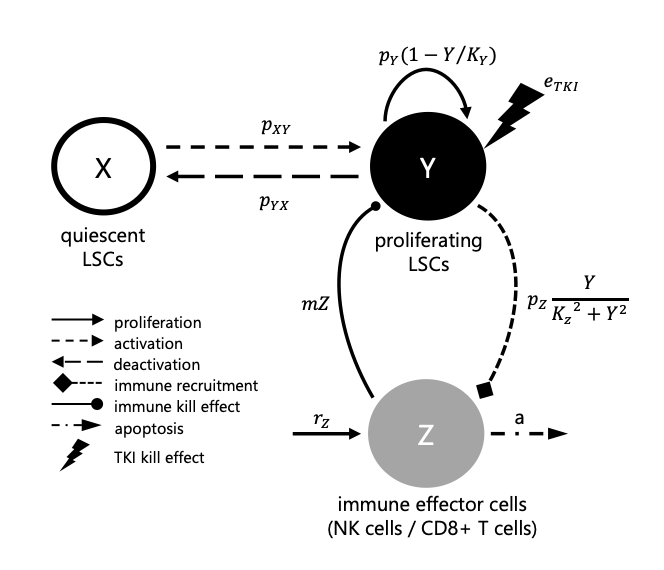

Supplement: S7 Fig — Leukemic stem cells (LSC) can reversibly change between two states X and Y (according to the rates pXY and pYX, respectively): X defines the quiescent, non-replicating cells, Y defines the active, proliferating cells. LSC in Y proliferate according to a logistic growth model with maximal proliferation rate pY and carrying capacity KY. The TKI-effect is described by a constant rate eTKI affecting the leukemic cells in Y. Immune cells in Z are activated by cells in Y (immune recruitment), following an immune window approach (see Supporting information). At the same time the immune cells kill proportional target cell in Y. Immune cells in Z are generated with rate rz and decay with rate a. (TIFF) [file pone.0256585.s007.tiff]
