# Supplementary material for: How to predict relapse in leukemia using time series data: A comparative in silico study
Source: PLoS One. 2021 Nov 15;16(11):e0256585. doi: 10.1371/journal.pone.0256585 (PMC8592437; doi:10.1371/journal.pone.0256585)
Supplement: S1 File — (PDF) [file pone.0256585.s008.pdf]

AML

Clinical data

To be able to generate data with the mechanistic AML models that were as similar as possible to real clinical data, we used a patient data set for comparison. The data published together with the mechanistic model was used [20]. 61 of these 275 patients relapsed within two years after therapy initiation. The data consists of time course measurements of the relative tumor load of NPM1-mutated patients, by qPCR measurements of the amount of NPM1-mut transcripts relative to the amount of the reference genes transcripts (ABL).

Mechanistic model

Our mechanistic model of the molecular disease dynamics of AML, already published in [20], describes the dynamics of healthy stem cells (*H*) and leukemia-initiating cells (*L*) in the bone marrow of an AML patient (schematic overview in Figure S6). Each cell can adopt one of the two differential states: a quiescent state (Q) and an active state (A). The cells can reversibly switch between states with transition rates $t^A_{l/h}$ and $t^Q_{l/h}$. Cells in active state (H_A/L_A) proliferate with proliferation rate $p_{l/h}$ and are sensitive to chemotherapeutic treatment with the kill rate *c*. Active cells can also differentiate into other states and therefore leave the two observed states. Individual chemotherapy schedules as well as patient-specific transition rates from quiescent to active state of the leukemic cells (t_l^A) and proliferative potential of leukemic cells (p_L) are taken into account to adapt the model to individual patients. The model equation are as follows:

$$\frac{dH_Q}{dt} = t_h^Q \cdot \left(1 - \frac{L_Q + H_Q}{K_Q}\right) \cdot H_A - t_h^A \cdot \left(1 - \frac{L_A + H_A}{K_A}\right) \cdot H_Q \quad (1)$$

$$\frac{dL_Q}{dt} = t_l^Q \cdot \left(1 - \frac{L_Q + H_Q}{K_Q}\right) \cdot L_A - t_l^A \cdot \left(1 - \frac{L_A + H_A}{K_A}\right) \cdot L_Q \quad (2)$$

$$\frac{dH_A}{dt} = t_h^A \cdot \left(1 - \frac{L_A + H_A}{K_A}\right) \cdot H_Q - t_h^Q \cdot \left(1 - \frac{L_Q + H_Q}{K_Q}\right) \cdot H_A + \left(p_h \cdot \left(1 - \frac{L_A + H_A}{K_A}\right) - c - d_h\right) \cdot H_A \quad (3)$$

$$\frac{dL_A}{dt} = t_l^A \cdot \left(1 - \frac{L_A + H_A}{K_A}\right) \cdot L_Q - t_l^Q \cdot \left(1 - \frac{L_Q + H_Q}{K_Q}\right) \cdot L_A + \left(p_l \cdot \left(1 - \frac{L_A + H_A}{K_A}\right) - c - d_l\right) \cdot L_A \quad (4)$$

For individual patient fitting we minimized the residual error between model and data using the *MultiStart* function and the *fmincon SQP* algorithm in MATLAB. The search space for parameter fitting was set as follows: for t_l^A between 0.01 and 0.99 and for p_l between 0.04 and 0.2. The model was initialized using the steady state of the model with not leukemia advantage (meaning all parameters are the same for leukemic and healthy cells) and starting with 60% healthy and 40% leukemic cells in each compartment.

The other parameter values were fixed as follows: $K_A = K_Q = 10^5$, $t_h^A = 0.01$, $t_h^Q = 0.2$, $t_l^Q = 0.002$, $p_h = 0.04$, $d_h = d_l = 0.033$, $c = 0.99$.

Artificial data

For data generation, we took the chemotherapy information obtained from real patients (i.e. timing and number of cycles) and combined them with fitted parameters from the AML patient set [20]. The parameters were slightly varied by adding a white noise (standard normal distributed with $\mu_P = 0$ and $\sigma_P = 0.01$). The following 5 data sets with 5000 time-courses each, half of them relapsing, half of them non-relapsing within two years, and a measurement period of 9 months were generated (Supplementary Table 1):

- Dense (D) data: one measure per week starting with the first day of chemotherapy.
- Dense-noisy (DN) data: added white noise ($\mu_D = 0$, $\sigma_D = 0.5$) to the log-scaled data from D.
- Sparse-noisy (SN) data: Measurement frequency was adjusted to the number and intervals of measurements in the real data. Therefore, the real data was divided into three intervals (first 3 months, second 3 months and third 3 months) and for each interval the number of measurements was counted and randomly sampled for each artificial patient. From the DN data, a weighted sample of this number of measurements was then taken using the histogram of the time points in each interval to weight the sampling.
- Artificial patient (AP) data: all values of SN below the detection limit of -5 [log10] were set to -5 [log10].
- Artificial scheme (AS) data: using DN measurement time points at the beginning of each therapy cycle and every 6 weeks thereafter. All values below detection limit were set to -5 [log10].

The leukemic burden, which is the main readout of the model, is calculated as the relative fraction of leukemic cells in both states with respect to the overall number of all cells in these states, i.e.:

$$leuk.\ burden\ [\%] = \frac{L_A + L_Q}{H_A + H_Q + L_A + L_Q} \cdot 100\%$$

The leukemic burden is rescaled by a factor of 100 to compare it to the patient's NPM1/ABL measurements [20]. Only patients which reached remission after therapy and did not relapse within the first 9 months were included in the data set. If the leukemic burden exceeded the relapse threshold of 1% at two years after treatment start, the time series was classified as a relapse.

Supplementary Table 1: Number of patients and average number of measurements for all AML datasets. The reduced number of patients in SN, AP results from exclusion of cases where no measurements were scheduled from our algorithm.

	number of patients	average measurements per patient
**clinical data**	275	3.8
**D**	5000	39
**DN**	5000	39
**SN**	4973	3.8
**AP**	4973	3.8
**AS**	5000	7

Generalized linear model

For training a generalized linear model (GLM), in more depth a logistic regression classifier, we derived 5 features. Two were taken from the previous description of the main characteristics of the time course in AML [19]: the elimination slope α, giving a measure of the decrease of leukemic burden during therapy, until the relapse threshold of 10^{-3} is reached and the minimal leukemic burden after therapy (n). The other three features were derived from a simple model describing the time course with a starting point (y_0) at the beginning of treatment, a linear decrease with slope *a* of the leukemic burden during the time of treatment and a linear increase with slope *b* during treatment-free periods (see S4 A Fig). The starting points (y_0) and the two slopes (*a* and *b*), together with the two characteristics were then handed to the GLM. When fitting the GLM using the parameters it became clear that two of them could be left out without losing accuracy. Therefore, the final GLM was fitted to predict the relapse based only on the minimal leukemic burden after therapy (*n*), the decreasing slope during therapy (*a*) and the increasing slope during treatment free periods (*b*). A 10-fold cross validation was used to estimate the variation of the estimated accuracy.

Neural Network

The neural network consists of a bidirectional long-short-term-memory (LSTM) layer with 32 hidden units (using ReLU activation), followed by a fully connected layer with 64 nodes with a ReLU activation and a single, sigmoid output layer.

We use a vector of log10 values of NPM1/ABL values as input. Missing values (i.e. no measurement available at this particular time point) were encoded as -1 and a respective masking-layer was introduced to the network. For the non-detectable datapoints, we used the detection limit for the clinical data (see above). The time points of chemotherapy were given as a second channel input in the case of AML.

To prevent overfitting, 10-fold cross validation was used. We saved the model with the highest validation (not training) accuracy. The entire training process was repeated 10 times on each dataset, to report a more robust estimate of the network performance as we regularly observed

numerical instabilities in the training process leading to failure of model fitting. Networks were trained with binary cross-entropy loss using the Adam optimizer with a learning rate of 10^{-3} and a learning rate decay of 10^{-5}. The size of the validation set was chosen as 15 % of the training set. We used a batch size of 128 and trained for 100 epochs using an early stopping checkpoint to stop the training if the validation loss did not decrease in 20 epochs.

CML

Clinical data

To generate in-silico CML patients as similar to real patients as possible, we utilized a cohort of 21 CML patients based on [28].

Each measurement can be either a detectable or an undetectable value. If a measure is detectable a corresponding BCR-ABL1 ratio on a log scale (LRATIO) is given. An undetectable measure is defined by a quantification limit (lQL) dependent on how much of the reference gene ABL1 was found in the sample. The more ABL1 is in the sample, the lower is the quantification limit.

For CML, typically a biphasic course in the time series is observed. At first, rapid decline symbolizes the fast clearing of the majority of tumor cells. This phase usually takes between 6 and 12 months. After this initial phase, a second phase with a moderate decline begins, symbolizing the slow tumor degradation in bone marrow. During the treatment the tumor load measurable in blood becomes lower, thus the number of non-detectable measures increases over time.

Mechanistic model

The mechanistic model simulating the CML cells consists of 3 compartments (Supplementary Figure 7). X defines the quiescent, non-replicating cells, Y defines the active, proliferating cells and Z defines the immune cells specific to the CML cells described by X and Y. Cells change from a quiescent state into an active state and vice versa with certain probabilities defined by the rates p_{XY} and p_{YX}, respectively. Furthermore, Y cells proliferate with a logistic growth defined by a maximal rate p_Y. The TKI-effect is described by a constant rate TKI killing the corresponding proportion of leukemic cells in Y. Immune cells in Z get activated by the number of active cells in Y. At the same time the immune cells kill proportional cells from Y depending on its number. However, the activation function of Z, depending on Y, defines a so-called immune window. In other words, depending on the parametrization immune effector cells are suppressed on very high and very low numbers of active leukemic cells. In between the immune cells rise and get effective [28]. The model is implemented in *Julia*. For this model we are using the default Julia ODE solver configured like follows: We assume a non-stiff problem and apply the *Tsit5* algorithm. During solving, an auto-detection for stiffness is applied and in case stiffness is detected, the solver is switched to the *Rosenbrock23* solver.

Mechanistic parameters

To fit the model to clinical and in-silico data, we used the following model parameters:

- maximum activation rate of Z (p_Z)
- immune window suppressing constant (K_Z)
- cell switch rate from X to Y (p_{XY})

- cell switch rate from Y to X (p_{YX})
- maximum activation rate of Y (p_Y)
- TKI kill rate (e_{TKI})
- initial tumor burden (*initRatio*)

The following model parameters were fixed among all mechanistic simulations:

- maximum number of tumor cells in Y ($K_Y = 10^6$)
- kill rate of active leukemic stem cells by immune cells ($m = 10^{-4}$)
- constant additive influx of immune cells ($r_Z = 200$)
- apoptosis rate of immune cells ($a = 2.0$)

Fitting Mechanistic Model to Data

We fitted the mechanistic parameters of all clinical as well as in-silico patients in R using a genetic algorithm from a modified R-package *rgenoud* (version 5.8-3.0). The specific configuration and the corresponding package can be found in the repository. The fitness function is defined as the sum of the distance of all measurements with the following rules:

(1) detectable measures: quadratic distance between patient data and in-silico data
(2) undetectable measures: left censoring of values meaning if the simulation value is higher than the given IQL value (see Clinical Data) we use a quadratic distance, in case it is equal or lower, we use a 0-distance.

Artificial data

We generated the in-silico parameters using copulas to sample from the fitted patient parameters, such that we receive a highly similar correlation structure:

(1) Using Copulae (R-package *copula* 1.0-0), describing a functional dependency between the marginals of multiple independent variables and their corresponding joint probability distribution, we:
    (a) create a normal Copula with the correlation matrix reflecting the correlations of the empirical random variables,
    (b) generate the random observations with the Copula targeting marginal uniform distributions,
    (c) and transform it into the observed empirical distribution.

From this, we sampled 5000 in-silico patient parameters, whereas half are relapsing and half are non-relapsing patients (Supplementary Table 2). Additionally, we are taking the information of the properties of cessation time, measurement noise, density and frequency of undetectable values from the patient data. As properties differ at specific treatment phases, e.g. sample frequency is usually higher in the beginning, we are using the following sampling time intervals: 0-6, 6-12, 12-24, 24-36, 36-(cessation time).

(1) Dense Data (D): This dataset represents the raw simulation data taking one measurement per month. All measurements are noise-less and detectable.
(2) Dense-noisy Data (DN): We introduce noise, assuming it is solely represented by the distance between clinical data and the corresponding simulation fits. Therefore, we create an empirical noise distribution (END) representing the distances between clinical data and corresponding simulation fits for each interval. Next, using kernel

density estimates, we add an error value for each data point in D and its correspondent interval sampling from END.

(3) Sparse-noisy (SN): Using the clinical data, we calculate for each time interval a distribution of the time of the first observation (DFO) and a distribution of time intervals (DTI) by taking the differences of consecutive time points. Then, given an in-silico patient of DN and an interval, a starting time (ST) from DFO within the corresponding interval is sampled. After, the cumulative sum of sufficiently many samples from DTI is added to ST until the interval border is exceeded. Finally, only the data points of the in-silico patient from DN are kept if they are within the sampled time quantities. Doing this for all in-silico patients of DN and intervals, SN is generated from DN. All samples are taken continuously by using kernel density estimates.

(4) Artificial Patient (AP): For each interval we calculate a probability that a measurement is not-detectable. These probabilities are then applied to the in-silico patients from SN to receive the final in-silico patient data.

(5) Artificial Scheme (AS): We are simulating patients with a scheme inspired by the DESTINY trial (Clark et al., 2019). Therefore, we set the TKI dose in the model to 50% of the original dose 12 months before TKI cessation. To generate this AS dataset, we started all over from (1) -(4) with the only difference in the time intervals. As patients in DESTINY during dose reduction are monitored very closely, the last time interval is further divided into two parts (period from month 36 of treatment until TKI dose reduction, period of reduced TKI dose).

In all our CML simulations, we classify whether an in-silico patient relapses by simulating 10 years ahead and check whether the tumor burden is above MR1.

Supplementary Table 2: Overview of in-silico CML data sets in comparison with clinical data

	**number of patients**	**average measurements per patient**	**average treatment time until cessation (months)**
**clinical data**	21	23.05	90.98
**D**	5000	92.51	92.51
**DN**	5000	92.51	92.51
**SN**	5000	23.19	92.51
**AP**	5000	23.19	92.51
**AS**	5000	28.80	92.64

Generalized linear model

To predict the relapse outcome, we trained a logistic regression classifier (GLM) using

- the first slope α,
- the corresponding intercept A,
- the second slope β,

- the corresponding intercept *B*,
- the standard deviation during the biexponential phase σ,
- the cessation time and
- the BCR-ABL1 ratio at cessation time,

whereas α, A, β, B and σ result from the corresponding biexponential fits. We used R's default stats package to train the GLM.

In the case of the artificial scheme AS, the additional predictors were added:

- the third slope γ during half dose,
- the corresponding intercept *C*,
- the first point in time of the half-dose and
- the BCR-ABL1 ratio at half dose time.

We allowed first-order interactions. However, resulting models were simplified based on the AIC until a minimum is reached. We used a 10-fold cross-validation.

Neural Network

We used mostly the same configuration as described above for the AML data. As input, we used the vector of log10 values of BCR-ABL values. In case the value was not detectable, we used the IQL (see above, Supporting Information: CML - Clinical Data). To account for the more complex dynamics in the CML case and the higher number of data points within one time-series we increased the maximum number of epochs to 2000 with an early stopping if the validation loss does not decrease for 100 epochs.
